# Return to Running After Extracorporeal Magnetotransduction and Electromagnetic Shockwave Therapy for Bone Stress Injuries: A Retrospective Case Series

**DOI:** 10.3390/bioengineering13101176

**Published:** 2026-10-09

**Authors:** Ryan L. Friedman, Violet E. Sullivan, Logan W. Gaudette, Amol Saxena, Michelle M. Bruneau, Adam S. Tenforde

**Affiliations:** 1 School of Medicine and Health Sciences, George Washington University, Washington, DC 20037, USA; 2 Department of Physical Medicine and Rehabilitation, Harvard Medical School, Spaulding Rehabilitation Hospital, Charlestown, MA 02129, USA; 3 College of Osteopathic Medicine, University of New England, Portland, ME 04103, USA; 4 Podium Podiatry & Sports Medicine, Palo Alto, CA 94301, USA

**Keywords:** bone stress injury (BSI), stress fracture, extracorporeal magnetotransduction therapy (EMTT), electromagnetic shockwave therapy (ESWT), return to running (RTR), return to sport, running, bone healing

## Abstract

Bone stress injuries (BSIs) are overuse injuries to bone that account for substantial time lost for athletes who are training and competing. Each injury varies in healing time based on factors including the Female and Male Athlete Triad (Triad), anatomical location, injury severity, and chronicity. While focused extracorporeal shockwave therapy (ESWT) has been evaluated for management of BSIs, extracorporeal magnetotransduction therapy (EMTT) has been proposed to have synergistic effects. We evaluated return to running in athletes with BSIs treated with combined ESWT and EMTT. Retrospective chart review identified 46 athletes with 50 BSIs (29 female, 17 male; mean age 30.5 ± 13.2 years; body mass index 22.0 ± 2.8 kg/m^2^). Each athlete was comprehensively evaluated and provided individualized management of all risk factors identified, including Triad, anatomical location, MRI grade, and chronicity (acute < 3 months; delayed/nonunion ≥ 3 months). Each athlete elected to receive weekly to biweekly focused electromagnetic ESWT and EMTT (mean values: 4.8 sessions; ESWT, 3424 pulses/session, mean energy flux density 0.41 mJ/mm^2^; EMTT: 80 mT, 8 Hz, 7826 pulses/session). Additional ESWT/EMTT were based on factors including persistent pain during return-to-run progression. Most BSIs were high grade (n=35); Triad risk was moderate in 21 athletes and high in 6. Forty athletes (87%) returned to pain-free running. Return to run was defined as the start of a patient-reported pain-free return-to-run progression. Among 39 athletes with documented dates, this occurred sooner for acute than delayed/nonunion BSIs (12.9 ± 5.8 vs. 23.8 ± 12.1 weeks, p=0.002). Differences by injury grade (p=0.18), Triad risk (p=0.97), and anatomical classification (p=0.26) were not statistically significant, although subgroup sizes were small. While we recognize limitations related to study design and the population studied, our findings suggest the addition of ESWT/EMTT combined with comprehensive management of athletes with BSI may allow for a high rate of return to running.

## 1. Introduction

Bone stress injuries (BSIs) are overuse injuries to bone commonly seen in military personnel and endurance athletes, particularly runners [1]. In normal conditions, microscopic damage to bone resulting from loading undergoes targeted repair and bone remodeling. A BSI results when microdamage accumulates faster than it can be repaired, and the injury exists on a continuum from early BSI with evidence of bone marrow edema on MRI, to advanced injuries demonstrating presence of fracture lines, referred to as stress fractures [2]. The diagnosis of BSI is made based on clinical history, examination findings of pain localized to bone, and commonly with radiographic confirmation. BSIs often result in significant time away from sport for athletes and may take up to 24 weeks to heal with high-grade imaging findings for high-risk anatomical locations [3]. Radiographic grading using magnetic resonance imaging (MRI) can correspond with injury severity and return to sport [4,5]. High-risk anatomical locations have been identified as the femoral neck, anterior tibial cortex, tarsal navicular, and fifth metatarsal and are associated with prolonged recovery and higher risk of delayed healing or need for surgery [6]. Athletes who develop a BSI often have multiple factors contributing to the injury, and each risk factor should be addressed to optimize healing [1,3,6,7,8,9]. The Female and Male Athlete Triad describes the interrelationship of energy availability, hormone function and bone mineral density [8,9]. A greater number of Triad risk factors may cumulatively increase the risk for BSI and result in prolonged time to return to sport [5,10,11,12,13].

Each athlete with a BSI should receive a comprehensive care plan, which includes identifying and treating each risk factor. Triad risk factors are important to identify and are primarily treated non-pharmacologically by optimizing nutritional intake to ensure adequate energy intake [5,10,11,12,13]. In the initial phases of treatment, each athlete with a BSI should be instructed to modify physical activity, often with a period of non-weight bearing or off-loading the site of injury to achieve pain-free status combined with physical therapy and gradual return to sport [1,6]. However, athletes may seek additional strategies to facilitate bone healing and pain relief. Extracorporeal shockwave therapy (ESWT) is a form of external energy that can produce acoustic waves applied to bone and soft tissue with goals to reduce pain and improve healing [14]. The literature on ESWT for bone healing is growing, with the strongest evidence supporting its use in fracture nonunion and delayed union. In a meta-analysis of 23 studies, 876 of 1200 long-bone nonunions healed following ESWT, corresponding to an overall union rate of 73% [15].

The current evidence for ESWT in the management of BSI in athletic populations is limited to a few publications. Smaller descriptive reports include 5 athletes, 10 male soccer players and 19 trainee ballet dancers with lower leg stress fractures treated with ESWT [16,17,18]. In a trial of 18 male soccer players with proximal fifth-metatarsal stress fractures, half received ESWT and the other half underwent intramedullary screw fixation [19]. Both groups produced similar healing and outcomes, with median return to play at 13 weeks after ESWT and 16 weeks after surgery. The largest published study to date reported on the use of electromagnetic ESWT in 40 runners with 41 BSIs combined with optimization of nutritional intake and addressing other risk factors [20]. The authors reported a majority (39 of 40, 98%) returned to pain-free running with longer mean time for recovery in delayed/nonunion BSI.

In addition to ESWT, electromagnetic stimulation has been investigated as a non-invasive strategy to enhance healing of injuries to bone. Much of the earlier clinical evidence uses Pulsed Electromagnetic Field Therapy (PEMF), which delivers magnetic-field strengths of 1–10 mT. PEMF was introduced clinically in the 1970s and received FDA approval for fracture nonunion in 1979 [21]. However, studies on PEMF for fracture nonunion have provided mixed results in improving both pain and function [22,23]. Clinical trials and systematic reviews of PEMF for acute fractures have similarly shown limited to no benefit in healing or pain reduction [24,25].

These inconsistent findings with conventional PEMF have prompted interest in newer electromagnetic devices, with greater field strength and more dynamic features. Electromagnetic Transduction Therapy (EMTT) devices produce field strengths that are 10× stronger than PEMF, delivering approximately 80–150 mT with oscillation frequencies exceeding 100 kHz [26]. In contrast to ESWT which delivers acoustic mechanical energy, EMTT generates a rapidly oscillating magnetic field. In vitro studies of human osteoblasts showed that EMTT increased expression of osteogenic transcription factors RUNX2 and SP7 and genes involved in collagen synthesis and mineralization [27]. These changes at the osteoblastic level may result in accelerated formation of mineralized matrix, the structural foundation for new bone formation. In mesenchymal stem cells, EMTT increased VEGF secretion, promoting the vascular response necessary for promoting injury repair [26].

While pre-clinical studies suggest the feasibility of using EMTT for the management of bone-related pathology, limited studies have described the clinical application and outcome in humans. In theory, the combined use of ESWT and EMTT may provide a synergistic effect on bone healing. Saxena et al. compared patients who received combined ESWT and EMTT following foot and ankle surgeries that included osseous structures with historical controls matched by surgery type who received neither treatment. Patients treated with EMTT and ESWT had observed a greater rate of early bone healing on radiographs, reported less postoperative pain upon weightbearing, and returned to activity sooner [28]. A series of single-patient case reports documented radiographic healing without surgery following EMTT and focused ESWT in the management of delayed calcaneal, humeral, and hamate nonunions [29,30,31]. Other studies have suggested the benefit in facilitating accelerated return to play following surgical management of a clavicle fracture in a triathlete and in post-operative management of nonunion of high tibial osteotomy [32,33]. To date, limited work has described the use of EMTT and ESWT in the management of BSI in running-based athletes. The purpose of this study was to characterize the treatment outcomes of combined EMTT and focused ESWT in athletes who received comprehensive management of BSI while evaluating whether there were observed differences in treatment outcomes by chronicity, anatomical location, grade of injury, and health factors of the Triad.

## 2. Materials and Methods

### 2.1. Study Procedures

We retrospectively reviewed the electronic medical records of a single sports medicine physician (A.S.T.) to identify athletes who received at least two treatment sessions with a combination of EMTT and focused ESWT for treatment of BSI in all consecutive patients between November 2022 and August 2025. Patients were included if they were ≥18 years of age, identified as an athlete, had a BSI confirmed on advanced imaging (MRI or CT) in the lower extremity excluding the hallux sesamoids, received both EMTT and electromagnetic ESWT for the index injury, and complied with the prescribed rehabilitation with sufficient follow-up to determine return to running.

There were 54 patients who met the criteria above. Of those, 8 were excluded due to other injuries or other medical conditions influencing return to running (6) and unavailable original imaging (2). This provided a final cohort of 46 athletes with 50 treated bone stress injuries. Four patients had two concurrently treated BSIs.

### 2.2. Treatment Protocol

Each athlete completed an initial evaluation, including history, physical examination, and review of the relevant imaging. Patients completed questionnaires with information regarding their primary sport, average weekly volume of running prior to injury, prior BSI, current or past history of an eating disorder, and for females, menstrual history (age of menarche and fewest number of menstrual cycles in a year). Each athlete received detailed management of BSI. Athletes were counseled on adequate calcium and vitamin D intake and referred to see a sports dietitian if concerned about low energy availability or multiple Triad risk factors. Additional testing, including dual-energy X-ray absorptiometry (DXA), was obtained using published criteria, and further laboratory tests were obtained to evaluate for factors contributing to low bone density [8,9].

The treatment management plan for each athlete was developed based on identified risk factors, and delivery of ESWT/EMTT was performed in the clinic of a single sports medicine physician (A.S.T.). As the procedures are not covered by insurance, a single out-of-pocket fee was charged to perform the series of electromagnetic ESWT and EMTT. Both electromagnetic ESWT and EMTT were administered together at the same visit in nearly all cases, and sessions were repeated at 1–2 week intervals. Electromagnetic ESWT was delivered using the Duolith (Storz Medical AG, Tägerwilen, Switzerland). Each treatment session delivered 1500 to 5000 pulses, with the energy flux density advanced as tolerated to a maximum of 0.55 mJ/mm^2^. The use of clinical focusing (treatment of the site of anatomical pain) was used to guide the focal zone for ESWT application. EMTT was delivered with a Magnetolith device (Storz Medical AG, Tägerwilen, Switzerland) at 80 mT and an impulse frequency of 8 Hz with 4000 to 14,000 pulses per session with the EMTT coil centered over the primary site of pain. No patient underwent sedation or anesthesia. Weight-bearing restrictions or use of immobilization were based on the anatomical location of injury or whether there was pain with ambulation. Tarsal navicular injuries were managed with a minimum of 6 weeks of non-weight bearing with crutches and a tall walking boot to limit plantar flexion. Each athlete was restricted from any activity that caused pain and provided instructions on individualized introduction of weight-bearing and physical therapy.

Patients returned for follow-up evaluation approximately 6 to 8 weeks after the initial session. The physical examination was repeated to evaluate for clinical healing and was specific to the anatomical location of injury. Exams typically included single- and double-leg hop tests and evaluation for pain with percussion and palpation of the affected bone. Repeat imaging was obtained at the physician’s discretion (A.S.T.) based on a combination of factors, including the presence of pain, the anatomical location, and whether there were clinical concerns for delayed healing. The decision to clear patients for a graded return-to-run progression or provide additional treatments of ESWT/EMTT was based on clinical decision-making of the treating provider. Each athlete was prescribed physical therapy and given clearance on timing to start plyometric exercises in preparation for return to running. Clearance for return to running was based on meeting clinical criteria for healing and progress in physical therapy in plyometrics and strength gains. Return-to-run progression involved either use of a walk–run program or an anti-gravity treadmill individualized to the athlete but typically starting at 60–70% of bodyweight and intervals of running advanced gradually (Alter-G, Fremont, CA, USA). Additional booster sessions of ESWT and EMTT were provided during return-to-run progression based on discretion of the treating physician and were recommended when concerns for delay in healing due to persistent pain presented.

### 2.3. Data Processing

All available imaging was reviewed by the treating physician (A.S.T.) and graded 1–4 using appropriate MRI classification systems [4,5,7].

Biological risk was scored as low, moderate or high, using the cumulative risk assessment (CRA) for the Female and Male Athlete Triad [8,9]. The scored factors are low energy availability with or without disordered eating, low body mass index, low bone mineral density, prior BSI, and reproductive dysfunction in females, including delayed menarche, oligomenorrhea, or amenorrhea.

Injuries were categorized by time from diagnosis to first treatment with ESWT and EMTT: acute (<3 months), delayed (3–6 months) and nonunion (>6 months) [34]. The diagnosis date was defined as the date of the confirming imaging. In the four patients with two BSIs, two patients had two acute injuries, and the other two had a delayed and a nonunion. In patients treated for two BSIs of differing MRI grade or anatomical risk, the higher grade/risk was used to classify the patient for grade-stratified analyses.

The anatomical locations (separated by low and high-risk), radiographic severity, and chronicity were characterized across patients and evaluated for time of medical clearance and return to running. Medical clearance was defined by the date of the in-person follow-up visit when patients were cleared to start progressive plyometric loading. Return to running was defined as the patient-reported date on which a pain-free return-to-run progression began. Time to return to running was calculated from the first combined ESWT and EMTT treatment session to this date. The six treatment failures are not included in the time to medical clearance or return to run, as they did not meet these objectives. Patients who returned to running without a documented date were included in the proportion who successfully returned to running but excluded from time-to-event analyses. A treatment failure was defined as a patient who underwent surgery for the index injury or did not return to pain-free running within the period they were followed. Inter-rater reliability of outcome extraction was not assessed.

### 2.4. Statistical Analysis

Statistical analyses were performed using R version 4.6.0 (R Foundation for Statistical Computing, Vienna, Austria). Return to running and number of treatments were set as the dependent variables, while chronicity, MRI grade, anatomical risk category, and Athlete Triad risk categories served as independent variables. The dependent variables were determined to be non-parametric using the Shapiro-Wilk test and visual inspection. For independent variables with two groups (chronicity, MRI grade, and anatomic risk category), the Mann–Whitney U test was used. For independent variables with more than 2 groups (Athlete Triad risk category), the Kruskal-Wallis test was used. Alpha was set at 0.05 for all analyses. Demographic and treatment variables are reported as means with standard deviation. Categorical data are reported as counts and percentages. The inclusion of all athletes receiving ESWT and EMTT, dates of treatment with sufficient time to evaluate clinical outcomes of interest, and the study design did not allow for an a priori sample-size calculation for outcomes based on sex, Triad risk factors, anatomical site, or chronicity. The cohort included all eligible patients treated during the study period. Comparisons by chronicity, MRI grade, anatomic risk, and Triad risk category were specified in advance. Bonferroni correction was applied for all comparisons.

## 3. Results

### 3.1. Patient Demographics

We identified 46 patients with 50 BSIs treated with ESWT and EMTT. The cohort was composed of 29 women and 17 men, with a mean age of 30.5 ± 13.2 years and a mean body mass index of 22.0 ± 2.8 kg/m^2^. Forty-three patients were identified as runners, 1 professional basketball player, 1 collegiate soccer player and 1 collegiate lacrosse player. Amongst those who identified as runners, pre-injury running volume averaged 66 ± 29 km per week. The distribution of runners included 6 high schoolers, 14 collegiate, and 4 professional, and the remaining 19 were recreational runners, primarily training or competing in the marathon distance.

### 3.2. Female and Male Athlete Triad Cumulative Risk Assessment

Athlete Triad Risk was scored for all patients. Nineteen (41%) were low risk, while 21 (46%) were moderate and 6 (13%) were high risk. Time for return to running was not statistically different across Triad risk categories (p=0.97). While not scored as part of the CRA, three men were found to have clinically low testosterone and another with GH deficiency.

### 3.3. Injury Characteristics

**Figure 1:**
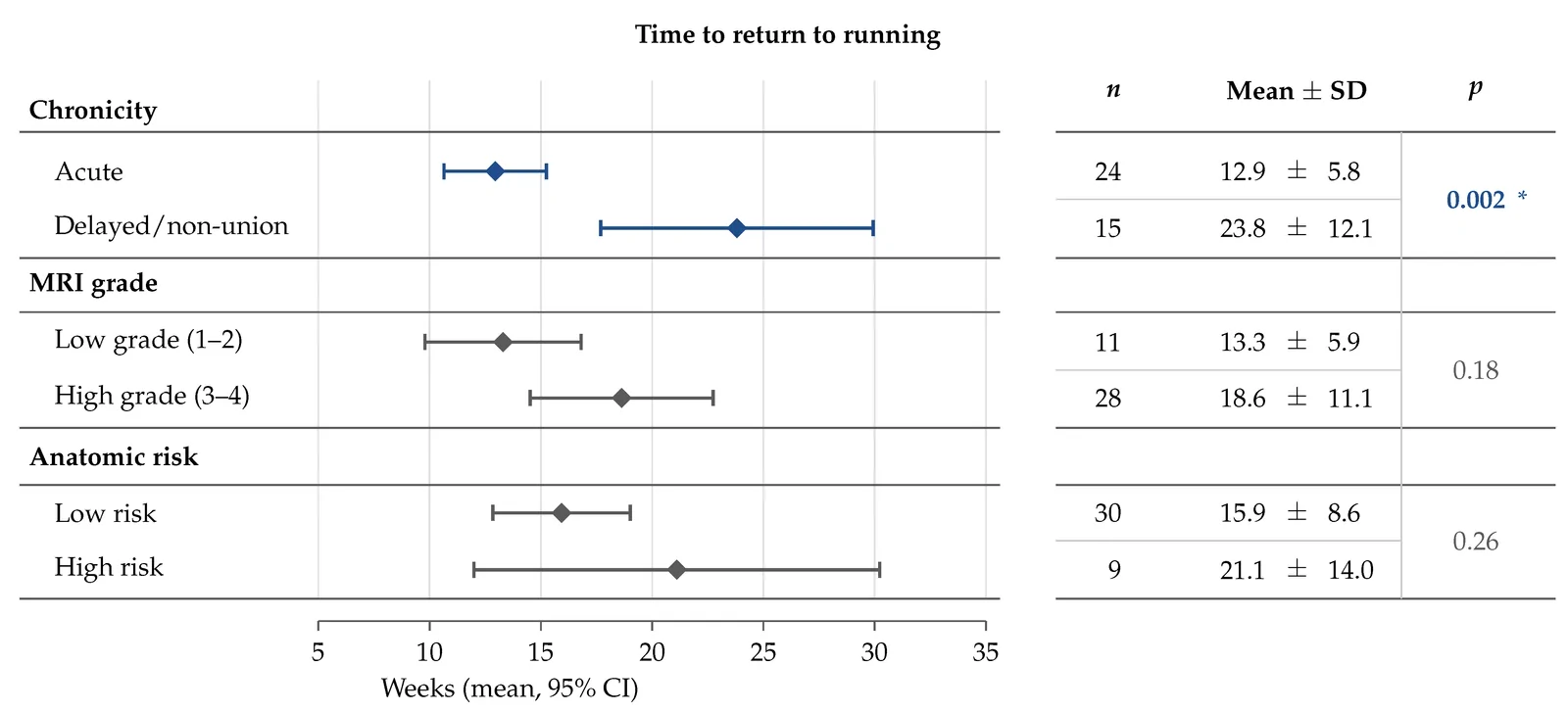
Time to return to running by injury chronicity, MRI grade, and anatomic risk category among the 39 patients with a documented return-to-run date. Diamonds represent group means, and horizontal bars are the 95% confidence intervals. Acute injuries returned at 12.9 ± 5.8 weeks compared with 23.8 ± 12.1 weeks for delayed union or nonunion (*p* = 0.002, denoted by *). Return to running did not differ significantly by MRI grade (13.3 ± 5.9 vs. 18.6 ± 11.1 weeks, *p* = 0.18) or anatomic location of low and high risk (15.9 ± 8.6 vs. 21.1 ± 14.0 weeks, *p* = 0.26).

The most common injury location was the tibia (n=20, 40%), followed by the metatarsals (n=11, 22%), and the full distribution of BSI by anatomical sites of injury is shown in Table 1. Group differences of acute BSI had shorter return to running than delayed/nonunion BSI (12.9 ± 5.8 vs. 23.8 ± 12.1 weeks, p=0.002). By anatomy, high-risk locations did not show a significantly longer time to return to running than low risk (Figure 1). Acute high-risk BSI returned at 16.6 ± 4.0 weeks (n=4) and low-risk BSI 12.2 ± 5.9 weeks (n=20); U=60.5, p=0.11. Delayed and nonunion high-risk BSI returned at a mean of 24.7 ± 18.5 weeks (n=5) and low-risk BSI at mean 23.3 ± 8.7 weeks (n=10); (p=0.59). Using MRI grading of BSI, fifteen injuries (30%) were low grade (1–2), and 35 (70%) were high grade (3–4). No measurable difference was observed in time for return to running between low- and high-grade BSI using MRI (13.3 ± 5.9 vs. 18.6 ± 11.1 weeks, n=11,28; U=110, p=0.18, Figure 1).

### 3.4. Treatment Characteristics

In addition to comprehensive management of risk factors, most athletes (n=42) had one BSI, and four were treated for two BSIs using ESWT/EMTT. Each anatomical location of BSI was treated with combined ESWT and EMTT. Thirty BSIs were categorized as acute, while 20 met the criteria for delayed/nonunion. The mean time from diagnosis to treatment was 30 days (range 1–88) for patients with an acute BSI and 248 days (range 94–890) for patients with delayed/nonunion BSI.

The total number of treatments per BSI was 4.8 ± 1.0 (initial 3.8 ± 1.0, booster 1.0 ± 1.4). For electromagnetic ESWT, patients received an average of 3424 ± 925 pulses per session with a mean of 0.41 mJ/mm^2^ (range 0.10 to 0.55). EMTT was delivered at a fixed intensity of 80 mT and frequency of 8 Hz, with a mean of 7826 ± 1450 pulses per session (range 4000 to 14,000). Most patients (n=36, 78%) also received radial pressure wave therapy targeting the affected soft tissue. There were no significant differences in total number of initial treatments or boosters based on chronicity or MRI grade (all p>0.05).

### 3.5. Radiographic Imaging

Twenty-seven patients (59%) had subsequent imaging after the initial course of ESWT and EMTT (Table 2). Eight patients had imaging prior to medical clearance, 14 had imaging during return-to-run progression, and five patients had repeat imaging and were ultimately classified as treatment failures due to persistent pain and limitations in return-to-sport. The imaging findings of healing were not consistently associated with a return to running pain-free.

### 3.6. Clinical Outcomes

Forty athletes (87%) returned to pain-free running. The mean time to medical clearance for progressive weight bearing was 6.3 ± 1.9 weeks for acute BSI (n=25) and 9.2 ± 3.6 weeks for delayed/nonunion BSI (n=15). The mean time to return to pain-free running was 12.9 ± 5.8 weeks (n=24) for acute BSI and 23.8 ± 12.1 (n=15) for delayed/nonunion BSI (Figure 1). On average, the return to run for acute BSI was shorter than chronic BSI (>3 months) (U=69, p=0.002). Of note, 14 athletes (30%) used an anti-gravity treadmill prior to land running, while the other 32 (70%) used a land-based walk–run progression.

One patient with an acute navicular BSI returned to running, but a specific date could not be established; this patient is included in the 40 who returned to pain-free running and in the time to medical clearance but is excluded from the time to return to running.

Four of the six athletes with treatment failures, defined as inability to return to pain-free running, elected for surgical management (2 navicular, 1 tibia, 1 metatarsal). Five of six were grade 4 BSIs. Five had follow-up imaging after ESWT/EMTT treatment, shown in Table 3. The four who elected surgery did eventually return to pain-free running. Two of the six failures did not undergo surgery. One was a delayed grade 2 metatarsal stress injury whose follow-up MRI showed it had healed; however, the patient had persistent intermittent pain and did not return to running. The other athlete had a grade 4 cuboid nonunion with follow-up imaging showing no healing and unchanged pain after treatment. There were no adverse complications reported by any patients with ESWT and EMTT.

## 4. Discussion

The purpose of this study is to describe clinical outcomes in athletes with BSI who received comprehensive management of risk factors and elected treatment with electromagnetic ESWT and EMTT. In our cohort, 40 of 46 athletes (87%) returned to pain-free running after management of their BSIs and addition of ESWT/EMTT. Injury chronicity was the only variable significantly associated with shorter return to run, with mean time for acute BSI associated with less time than delayed or nonunion BSI (12.9 ± 5.8 vs. 23.8 ± 12.1 weeks). MRI grade, anatomical risk classification, and the Female and Male Athlete Triad Cumulative Risk Assessment categories were not associated with statistically significant differences in time to return to running, although these subgroup comparisons were limited by small numbers.

Higher MRI grade and high-risk anatomical locations have each been established as predictors of prolonged recovery in BSI [3,7]. While we did not detect measurable differences, the mean return to running duration trended towards longer times with more advanced MRI grades (11.6, 18.0, 17.7, and 19.1 weeks for grades 1 through 4), consistent with the grade-dependent pattern reported in a large systematic review (6, 10, 14, 16.1 weeks) [7]. However, the longer time for return to sport across grades was less pronounced in our cohort, a 65% rise from grade 1 to grade 4 versus 135% in the prior meta-analysis [7]. While the study design limits definitive conclusions and the management of risk factors for BSI remains the primary strategy for treatment, our findings may suggest a role for adding ESWT/EMTT in higher-grade BSIs.

The Female and Male Athlete Triad Cumulative Risk Assessment is associated with risk for BSI, with higher scores indicating a greater likelihood of injury and prolonged return to sport [8,9,10,11]. Fifty-nine percent of patients scored as moderate- or high-risk Triad. However, time to return to running did not differ significantly by Triad risk category. Notably, each athlete was evaluated and treated for these risk factors as part of comprehensive management of BSI. This includes counseling on adequate energy availability, ensuring calcium intake primarily from diet, vitamin D supplementation, and iron supplementation when appropriate. Management by a multidisciplinary team was recommended for those with disordered eating/eating disorders, and hormonal disruptions (such as amenorrhea or low testosterone) were queried and targeted for treatment as these risk factors are critical to address in the management of BSI [8,9,35]. A subsequent BSI was noted in 11 athletes (24%) in the follow-up period. The average period of follow-up was 11 months. Ten athletes sustained a BSI at a different anatomical site, and one recurred at the same site. This is consistent with the known risk of prior BSIs being a strong predictor of future BSIs [11,36]. This recurrence highlights the complexity of treating these athletes. Management should include a multidisciplinary approach addressing nutritional, hormonal, biomechanical, training-related, and psychological contributors to prevent future injuries.

The most comparable published series of BSI described 40 runners with 41 bone stress injuries managed using the same electromagnetic ESWT combined with comprehensive management and reported that 39 of 40 returned to pain-free running [20]. Our return to pain-free running of 87% may be best explained by a higher complexity cohort rather than by treatment effect. This cohort had more delayed union or nonunion injuries (40% vs. 29%), more high-grade BSIs (70% vs. 49%), and high-risk locations (26% vs. 22%), particularly the navicular (6 vs. 1). Navicular BSIs are particularly difficult to treat. A meta-analysis found them to have the longest average recovery time of 127 days and a complication rate of 24% [3]. We observed similar outcomes, as navicular BSIs accounted for two of six failures and the single longest return to running of 55 weeks.

Our treatment protocol aligns with the recent Delphi study on shockwave therapy that recommended the use of high-energy settings of ESWT as being the most appropriate when applied for the management of BSI. The experts in this Delphi consensus recommended a minimum of three to four sessions at one- to two-week intervals using high-energy settings [14]. Our cohort received a mean of 4.8 ESWT sessions and energy flux density was titrated to a mean of 0.41 mJ/mm^2^ with most athletes achieving high energy settings (defined as EFD 0.30 mJ/mm^2^). Notably, each athlete with BSI was managed using best practice guidelines for comprehensive management of each risk factor [1], and a separate Delphi on BSI in athletes did not conclude that ESWT was an effective treatment [6]. Therefore, we recommend further reassessment if clinical improvement is not noted at the appropriate time after five treatment sessions of ESWT/EMTT.

An additional observation was that follow-up imaging did not always align with clinical recovery. Several patients continued their run progression pain-free while imaging still showed residual findings, including cortical thickening and extensive marrow edema that was not significantly changed from prior imaging. Conversely, one patient demonstrated radiographic healing but, due to persistent pain, did not return to pain-free running during the time of clinical management. The diagnosis of a BSI is based on clinical history and often imaging to confirm the presence of BSI [2,6]. However, bone marrow edema can be present in asymptomatic athletes, which limits current use of routine imaging to determine healing status [37,38,39]. This current study suggests that radiographic imaging may not always correspond with other clinical features of BSI healing.

There is no standard protocol for clinical use of EMTT, and the identified published studies did not describe the same device settings for treating osseous conditions. A controlled trial in degenerative joint and tendinopathy injuries used eight sessions of EMTT monotherapy delivered once weekly at 80 mT, 8 Hz, and 10,000 pulses [40]. A series of case reports on humeral, hamate, and calcaneal nonunion or delayed union and a case pilot study following foot and ankle surgery used three to five EMTT sessions at 80 mT, 6–8 Hz and 4000–6000 pulses [28,29,30,31]. Our cohort received treatment at 80 mT and 8 Hz with 4000 to 14,000 pulses per session. Developing protocols to determine the effective frequency and treatment settings may help to optimize use of EMTT in BSI.

No adverse events were reported during treatment sessions in the cohort. Patients typically experienced a transient increase in pain during ESWT treatment, which resolved without intervention. Pain was not typically reported during EMTT.

### Study Strengths and Limitations

To our knowledge this is the largest study to date on combined ESWT and EMTT for the treatment of BSIs in athletes. Strengths of this study include the large number of high-grade, anatomically high-risk, delayed/nonunion BSIs in athletes primarily with elevated Triad risk, allowing us to evaluate treatment in arguably more complex patients referred to the provider due to expertise in management of BSI and bone health (A.S.T.). All athletes were evaluated and treated by the same sports medicine physician, limiting inter-provider variability in treatment delivery and time to medical clearance. The large majority of athletes also completed rehabilitation within the same clinical system, providing greater consistency for rehabilitation. Follow-up imaging was available in a substantial number of patients, providing additional information beyond clinical symptoms. Limitations of the study include its retrospective design, patient-reported outcomes, and the lack of a control group. Additionally, we cannot account for the role of ESWT/EMTT over optimal treatment of BSI through comprehensive risk factor assessment and management of loading, physical therapy, nutrition, and Triad factors, which influence healing. The number of athletes with different Triad risk factors, MRI grades, and anatomical locations classified as high vs. low risk limited the ability to understand how each factor might influence outcomes of treatment. Further, this study design does not allow for comparing outcomes due to natural history or placebo/sham treatment. The contributions of ESWT and EMTT, individually or together, cannot be determined. Treatment was not standardized, as the number and delivery of treatment sessions varied based on clinical response, provider judgment, and logistical factors. Small subgroup sizes limited statistical power, and nonsignificant findings do not exclude clinically important differences. Finally, we included a younger athlete population with goals to return to running, and it’s unclear whether these results would apply to other populations such as the military, non-athletes, athletes in upper extremity sports, or those with older ages.

Our results suggest that ESWT/EMTT was safe, and a large number of athletes were able to return to running. Future research evaluating ESWT and/or EMTT in the management of acute BSIs through placebo-controlled randomized clinical trials may help clarify our findings.

## Figures and Tables

**Table 1:** Demographic and injury characteristics of the study cohort (46 patients, 50 bone stress injuries).

**Category**	**Characteristic**	**%**	***n***
Sex ^1^	Female	63%	29
	Male	37%	17
Primary sport ^1^	Running	93%	43
	Basketball	2%	1
	Soccer	2%	1
	Lacrosse	2%	1
Level of competition ^1^	High school	13%	6
	Collegiate	35%	16
	Professional	11%	5
	Recreational	41%	19
Athlete Triad risk score ^1^	Low	41%	19
	Moderate	46%	21
	High	13%	6
Location ^2^	Tibia	40%	20
	Metatarsal	22%	11
	Navicular	12%	6
	Femur	12%	6
	Pelvis/sacrum	6%	3
	Fibula	4%	2
	Talus	2%	1
	Cuboid	2%	1
MRI grade [4,5] ^2^	Low (1–2)	30%	15
	High (3–4)	70%	35
**Characteristic**	**Units**	**Mean**	**SD**
Age	years	30.5	±13.2
Body mass index (BMI)	kg/m^2^	22.0	±2.8
Pre-injury training volume ^3^	km/week	66	±29

^1^ Percentages of 46 patients. ^2^ Percentages of 50 bone stress injuries. ^3^ Among patients whose primary sport was running (n=43). Percentages are rounded and may not sum to 100.

**Table 2:** Follow-up imaging obtained after the initial treatment series in patients who returned to running. Time is reported in weeks from the initiation of EMTT.

Timing	Method	*n*	Mean ± SD (Weeks)	Range (Weeks)	Findings
Prior to medical clearance	MRI	5	6.8 ± 3.3	5–13	interval resolution of BSI, no edema (*n* = 2) interval healing with decreasing edema (*n* = 2) persistent low-grade (grade 1) stress reaction (*n* = 1)
	CT	3	8.0 ± 2.6	5–10	healed (*n* = 2) persistent fracture line (*n* = 1)
During return to run	MRI	12	17.3 ± 8.8	7–33	healed/resolved (*n* = 7) near resolution/resolving/improved with reduced edema (*n* = 3) residual cortical thickening without edema (*n* = 1) extensive marrow edema, similar to prior (*n* = 1)
	CT	1	32		no fracture line visualized, periosteal reaction present
	XR	1	22		no fracture

**Table 3:** Follow-up imaging in five treatment failures. Time is reported in weeks from the initiation of EMTT.

Method	*n*	Mean ± SD (Weeks)	Range (Weeks)	Findings
MRI	2	25.0 ± 2.8	23–27	healed (*n* = 1), persistent marrow edema with new subchondral edema (*n* = 1)
CT	2	27.0 ± 22.6	11–43	sclerotic persistent non-union (*n* = 2)
XR	1	8		incomplete healing

## Data Availability

The data supporting the findings of this study are available from the corresponding author upon reasonable request. The data are not publicly available due to privacy and ethical restrictions.
